# CXCR3 from chemokine receptor family correlates with immune infiltration and predicts poor survival in osteosarcoma

**DOI:** 10.1042/BSR20192134

**Published:** 2019-11-12

**Authors:** Yin Tang, Zhiqian Gu, Youwei Fu, Junjie Wang

**Affiliations:** Department of Orthopedics, HwaMei Hospital, University Of Chinese Academy Of Sciences, Ningbo 315000, Zhejiang, China

**Keywords:** chemokine receptors, CXCR3, immune, osteosarcoma, prognosis

## Abstract

**Background:** Chemokine receptors have a crucial role in regulating tumor mediating immunity and are also implicated in the prognosis of some cancers. Here, the association between CXC chemokine receptors (CXCR2–5) and prognosis in osteosarcoma was studied.

**Methods:** Differences between CXCR2, CXCR3, CXCR4, and CXCR5 expression and overall survival (OS) and event-free survival (EFS) were compared using Kaplan–Meier analyses. The associations of CXCR3 expression with clinical features and the prognosis were also analyzed. The signaling pathways modulated by CXCR3 were investigated. The correlations between CXCR3 and immune infiltrates were investigated.

**Results:** The expression of CXCR2, CXCR4, and CXCR5 was not associated with the prognosis, but CXCR3 low expression was correlated with worse OS and EFS of osteosarcoma, especially for female, patients aged less than 15.1 years, or patients without metastasis. Low CXCR3 expression was related to tumor site and histologic response (*P*<0.05), but not associated with other clinical characteristics. Multivariate Cox analysis revealed that CXCR3 remained independently associated with the prognosis, especially for OS (hazard ratio (HR) = 3.26, 95% CI = 1.15–9.24, *P*=0.026). The cell adhesion, apoptosis, metabolism, KRAS, P53, NOTCH, reactive oxygen species (ROS), PI3K/Akt/mTOR, vascular endothelial growth factor (VEGF), inflammation, and immune-related pathways such as IL-6/JAK/STAT3, TNF-α via NF-κB, Toll/NOD-like receptor, and complement were modulated by CXCR3. CXCR3 expression showed an especially positive correlation with immune infiltration of T cells CD8, macrophages M1, plasma cells, and NK cells activated.

**Conclusions:** CXCR3 may be an independent risk factor for the prognosis and is most likely to benefit from immunotherapy in osteosarcoma.

## Introduction

Osteosarcoma is the most common malignant bone tumor that occurs predominantly in young people and adolescents [1]. It accounts for approximately four to five cases per million people every year [2]. In recent years, osteosarcoma patients are treated by advanced surgery and combinational chemotherapy; the 5-year survival rate of non-metastatic osteosarcoma patients has increased to ∼60% [3]. Patients with metastasis or recurrence have a worse survival rate (20–30%) [4]. Therefore, more effective therapeutic strategies are urgently needed for the treatment of patients with osteosarcoma.

Chemokines and their receptors are involved in the recruitment, activation, and differentiation of immune cells [5]. They also play roles in angiogenesis, the attraction of leukocytes to tumor sites and induction of tumor cell migration, endothelial cell activation, and proliferation [6,7]. According to the presence or absence of glutamic acid-leucine-arginine sequence (‘ELR’ motif), the CXC chemokines are divided into two groups: ELR+ and ELR− chemokines [8]. CXC chemokine receptor 1 and 2 (CXCR1 and CXCR2) belong to the receptors of ELR+ chemokines, CXCR 3–6 are the receptors of the ELR− chemokines [9,10]. Among them, we mainly focused on the role of CXCR3 in osteosarcoma in our study. CXCR3 plays a role in diverse cellular functions such as chemotactic migration, cell proliferation, cell adhesion and invasion [11,12]. CXCR3 also regulates multiple signaling pathways, including the Ras/ERK, Src, and PI3K pathways [12]. Studies have reported the relationship of CXCR3 with the prognosis in many cancers. Patients with low CXCR3 expression show worse prognosis than patients with high CXCR3 expression in clear cell renal cell carcinoma and gastric cancer [8,13]. High CXCR3 expression is associated with poor survival in glioblastoma and colorectal cancer [14,15]. However, the relationship between CXCR3 and the prognosis in osteosarcoma has not been studied.

Based on the available data of CXCRs in osteosarcoma, we assessed the prognostic role of CXCR2, CXCR4, and CXCR5 expression in osteosarcoma. Then, the objective of the present study was to extensively evaluate the correlation between CXCR3 and the clinicopathological features and the prognosis in osteosarcoma. We further explored insight into the biological pathways modulated via CXCR3 and investigated whether CXCR3 was associated with immune infiltration in osteosarcoma.

## Materials and methods

### Sample information

The normalized expression data (Transcripts Per kilobase Million: TPM values) for patients with osteosarcoma were obtained from the Therapeutically Applicable Research to Generate Effective Treatments (TARGET). To remove genes with low expression, the mean expression levels with TPM > 1 in all samples were selected for genes. Finally, the mRNA expression profiles of the following chemokine receptor genes were available: CXCR2, CXCR3, CXCR4, and CXCR5. Clinical information was also obtained, including age, gender, tumor site, tumor region, surgery type, progression, histologic response, and distant metastasis. Finally, 98 osteosarcoma patients were enrolled between 2000 and 2012. The expression data of 98 osteosarcoma cases and clinical data were obtained in the present study. Overall survival (OS) (death) and event-free survival (EFS) (progression, recurrence, second malignant neoplasm, or death) were defined.

### Gene set enrichment analysis identifies a CXCR3-related signaling pathway

Gene set enrichment analysis (GSEA) was utilized to identify the potential biological mechanisms between two biological states [16]. The gene sets were collected from the Molecular Signatures Database (MSigDB) (‘hallmark (h.all) and c2.cp.KEGG.v6.2.symbols’). GSEA (3.0) was applied to explore the potential biological processes and signaling pathways of CXCR3 on the impact of osteosarcoma prognosis. Gene set permutations with 1000-times were conducted to acquire the normalized enrichment score (NES). The nominal *P*-value of less than 0.05 and false discovery rate (FDR) of less than 0.25 were used to quantify statistically significant enrichment.

### Tumor-infiltrating immune cells

CIBERSORT applied a deconvolution algorithm to estimate the proportions of tumor-infiltrating immune cells in the tumor microenvironment (TME). The CIBERSORT method was used to analyze the abundance of 22 types of infiltrating immune cells based on normalized expression data in cancer [17]. All osteosarcoma samples were analyzed for immune cell profiles using CIBERSORT, and the number of permutations with 100 was set. Osteosarcoma samples with a CIBERSORT *P*-value of less than 0.05 were selected and included in the present study.

### Statistical analysis

All statistical analyses were conducted by using R software (version 3.5.1; R Foundation for Statistical Computing, Vienna, Austria). The association between CXCR3 expression and tumor-infiltrating immune cells was estimated by Spearman’s correlation coefficient. The cut-off values were determined by their median values (CXCR2: 0.42, CXCR3: 0.51, CXCR4: 33.74, and CXCR5: 0.52); subsequently, each sample was divided into high expression group and low expression group. The Kaplan–Meier survival analyses with the log-rank test were utilized to compare differences between the low- and high-expression groups. The logistic regression analysis was applied to assess the relationship between CXCR3 expression and the clinicopathological characteristics of osteosarcoma. The univariate and multivariate survival analyses (hazard ratio: HR; 95% confidence interval: 95% CI) were performed with the Cox proportional hazards models to verify the associations between CXCR3 expression and the prognosis along with other clinical factors such as histologic response and metastasis. Additionally, according to the subgroups of age, gender, and metastasis status, we further explored the correlation of CXCR3 expression with the prognosis in various clinicopathological features.

## Results

### Basic patient characteristics

The clinical data from a total of 98 cases with osteosarcoma and the corresponding expression data of CXCR2, CXCR3, CXCR4, and CXCR5 were included. Clinical information included age, gender, tumor site, tumor region, surgery type, progression, histologic response, and metastasis (Supplementary Table S1). The median age at initial pathological diagnosis for osteosarcoma was 15.1 years, with a range from 3.6 to 39.9 years. Median follow-up time for EFS was 20 months, ranging from 0 to 143.8 months. Median follow-up time for OS was 37.4 months, ranging from 0 to 192 months.

### Kaplan–Meier analyses of CXCR2, CXCR3, CXCR4, and CXCR5 expression

**Figure 1:**
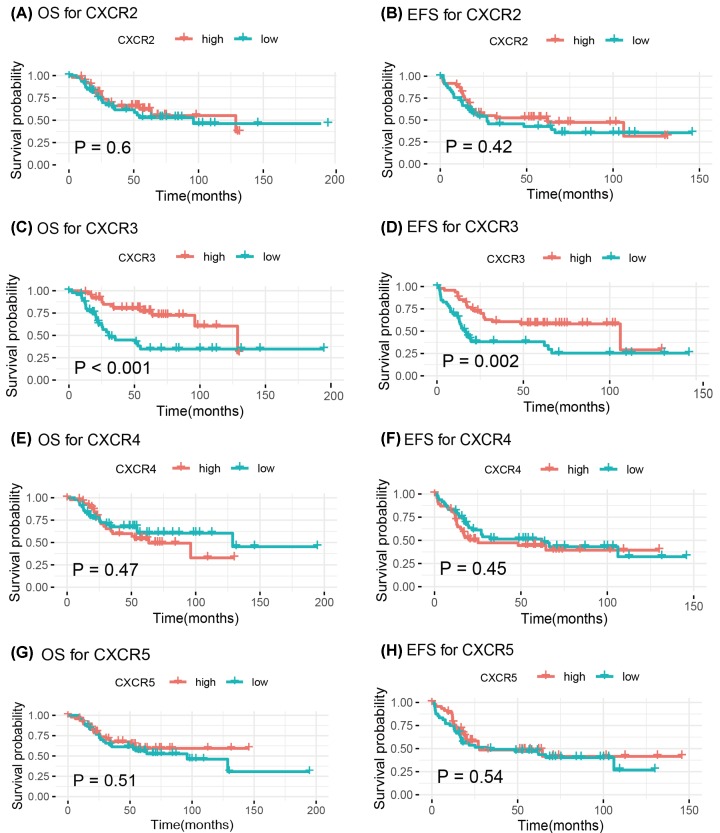
Kaplan–Meier curves for the associations of CXCR2, CXCR3, CXCR4, and CXCR5 expression with OS and EFS in patients with osteosarcoma (**A**) OS for CXCR2; (**B**) EFS for CXCR2; (**C**) OS for CXCR3; (**D**) EFS for CXCR3; (**E**) OS for CXCR4; (**F**) EFS for CXCR4; (**G**) OS for CXCR5; (**H**) EFS for CXCR5.

The Kaplan–Meier analyses were conducted to estimate the survival impact of expression of CXCR2, CXCR3, CXCR4, and CXCR5 in osteosarcoma (Figure 1). The expression of CXCR2, CXCR4, and CXCR5 were not associated with the prognosis in OS and EFS (all *P*-values >0.05) (Figure 1). Osteosarcoma with low CXCR3 expression showed an unfavorable prognosis than that with high CXCR3 expression (OS: *P*=0.00082; EFS: *P*=0.0022) (Figure 1C,D).

### Association of CXCR3 expression with clinicopathological variables

Only CXCR3 expression was related to the prognosis. Thus, the relationships of CXCR3 expression with the clinicopathological features were performed using the logistic regression analysis in 98 patients (Table 1). Low CXCR3 expression was not significantly associated with age, gender, tumor region, surgery type, progression, and metastasis, but was correlated with tumor site (femur vs. tibia: OR = 3.1, 95% CI = 1.02–9.37, *P*=0.046) and histologic response (poor vs. good: OR = 4.2, 95% CI = 1.21–14.54, *P*=0.024).

**Table 1:** Correlation of low CXCR3 expression with clinicopathological characteristics of osteosarcoma

Factors	*n*	OR (95% CI)	*P*
Gender (male vs. female)	98	1.4 (0.62–3.15)	0.412
Age (≥15.1 vs. <15.1 years)	98	0.78 (0.35–1.73)	0.545
Tumor site (femur vs. tibia)	68	3.1 (1.02–9.37)	0.046
Tumor region (distal vs. proximal)	59	1.19 (0.42–3.36)	0.746
Surgery (limb sparing vs. amputation)	54	2 (0.33–11.97)	0.448
Histologic response (poor vs. good)	52	4.2 (1.21–14.54)	0.024
Progression (yes vs. no)	48	2.29 (0.68–7.7)	0.182
Metastasis (yes vs. no)	98	2.48 (0.95–6.52)	0.064

Abbreviation: OR, odds ratio.

### Survival outcomes using univariate and multivariate analyses

The univariate Cox analysis demonstrated that decreased CXCR3 expression was correlated with worse OS (HR = 3.01, 95% CI = 1.53–5.92, *P*=0.001), along with the poor histologic response (*P*=0.028) and metastasis (*P*<0.001). After adjusting for the poor histologic response and metastasis, multivariate Cox analysis further showed that CXCR3 expression was an independent prognostic factor for OS (HR = 3.26, 95% CI = 1.15–9.24, *P*=0.026) (Table 2).

**Table 2:** Univariate and multivariate Cox analyses of OS in osteosarcoma

Variables	HR (95% CI)	*P*
Univariate analysis		
CXCR3 (low vs. high)	3.01 (1.53–5.92)	0.001
Gender (male vs. female)	0.97 (0.51–1.87)	0.931
Age (≥15.1 vs. <15.1 years)	0.88 (0.47–1.67)	0.7
Metastasis (yes vs. no)	3.81 (1.99–7.29)	<0.001
Tumor site (femur vs. tibia)	2.79 (0.96–8.16)	0.061
Tumor region (distal vs. proximal)	2.69 (1.00–7.25)	0.051
Surgery (limb sparing vs. amputation)	1.49 (0.35–6.42)	0.591
Progression (yes vs. no)	1.84 (0.86–3.92)	0.116
Histologic response (poor vs. good)	3.93 (1.16–13.36)	0.028
Multivariate analysis		
CXCR3 (low vs. high)	3.26 (1.15–9.24)	0.026
Metastasis (yes vs. no)	4.32 (1.61–11.63)	0.004
Histologic response (poor vs. good)	4.72 (1.22–18.21)	0.024

The univariate Cox model showed that low CXCR3 expression was associated with worse EFS (HR = 2.45, 95% CI = 1.38–4.35, *P*=0.002), and further multivariate Cox analysis demonstrated that CXCR3 expression was not significantly associated with EFS (HR = 4.45, 95% CI = 0.82–24.28, *P*=0.085) (Table 3).

**Table 3:** Univariate and multivariate Cox analyses of EFS in osteosarcoma

Variables	HR (95% CI)	*P*
Univariate analysis		
CXCR3 (low vs. high)	2.45 (1.38–4.35)	0.002
Gender (male vs. female)	1.25 (0.70–2.21)	0.453
Age (≥15.1 vs. <15.1 years)	0.67 (0.38–1.17)	0.161
Metastasis (yes vs. no)	2.68 (1.50–4.80)	<0.001
Tumor site (femur vs. tibia)	2.53 (1.10–5.83)	0.029
Tumor region (distal vs. proximal)	2.64 (1.17–5.94)	0.019
Surgery (limb sparing vs. amputation)	0.89 (0.31–2.57)	0.827
Progression (yes vs. no)	3.41 (1.71–6.81)	<0.001
Histologic response (poor vs. good)	4.50 (1.55–13.08)	0.006
Multivariate analysis		
CXCR3 (low vs. high)	4.45 (0.82–24.28)	0.085
Metastasis (yes vs. no)	1.74 (0.35–8.52)	0.497
Tumor site (Femur vs. Tibia)	5.86 (0.51–66.73)	0.154
Progression (yes vs. no)	2.97 (0.64–13.80)	0.164
Histologic response (poor vs. good)	1.05 (0.16–6.77)	0.963

### Correlation of low CXCR3 expression with the prognosis in various clinicopathological characteristics

**Figure 2:**
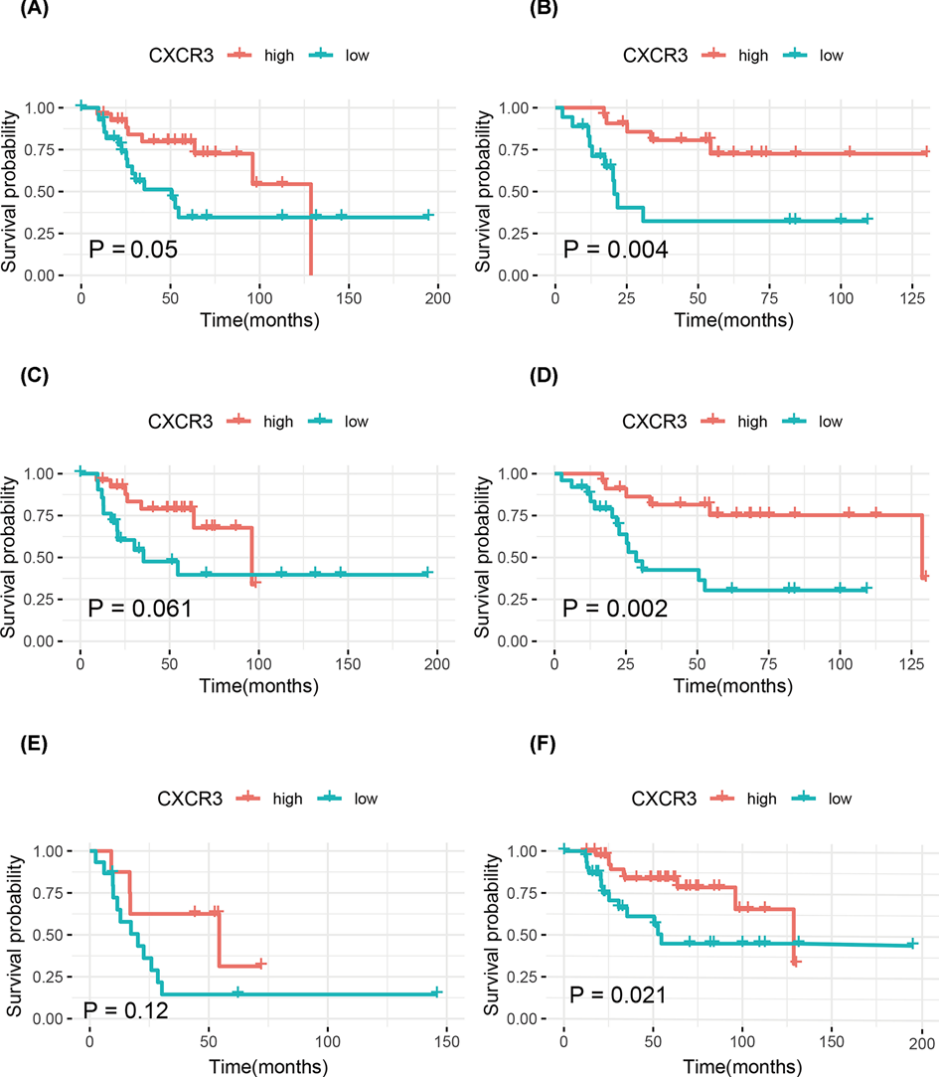
Kaplan–Meier curves for OS in osteosarcoma (**A**) Males; (**B**) females; (**C**) ≥15.1 years; (**D**) <15.1 years; (**E**) patients with metastasis; (**F**) patients without metastasis.

**Figure 3:**
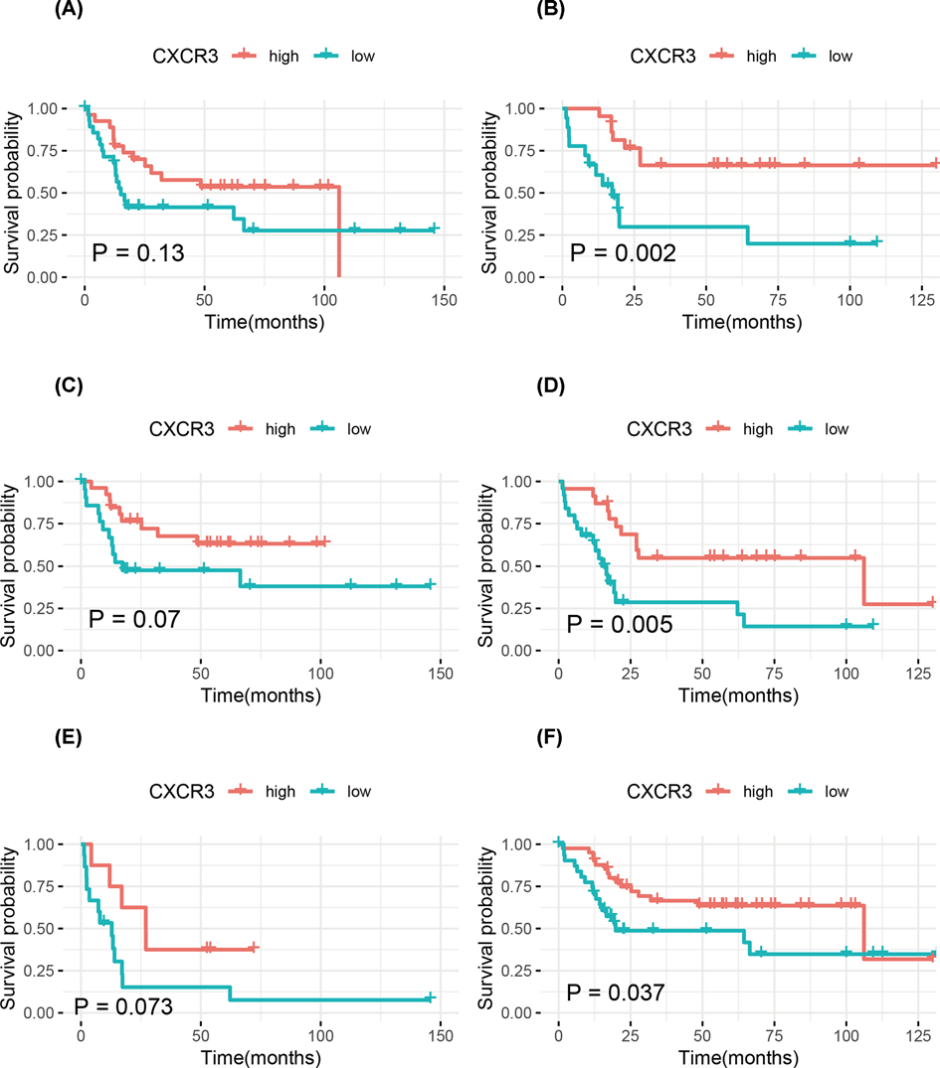
Kaplan–Meier curves for EFS in osteosarcoma (**A**) Males; (**B**) females; (**C**) ≥15.1 years; (**D**) <15.1 years; (**E**) patients with metastasis; (**F**) patients without metastasis.

The association of low CXCR3 expression with the prognosis were further explored in various clinicopathological features, such as age, gender, and metastasis status of osteosarcoma patients. Patients with the other clinicopathological characteristics were removed from the stratification analysis due to the small sample sizes. Kaplan–Meier curves showed that patients with low CXCR3 expression had significantly worse OS and EFS than those with high CXCR3 expression for female, patients aged less than 15.1 years, or patients without metastasis (all *P*-values <0.05) (Figures 2 and 3).

### The signaling pathways modulated by CXCR3

**Figure 4:**
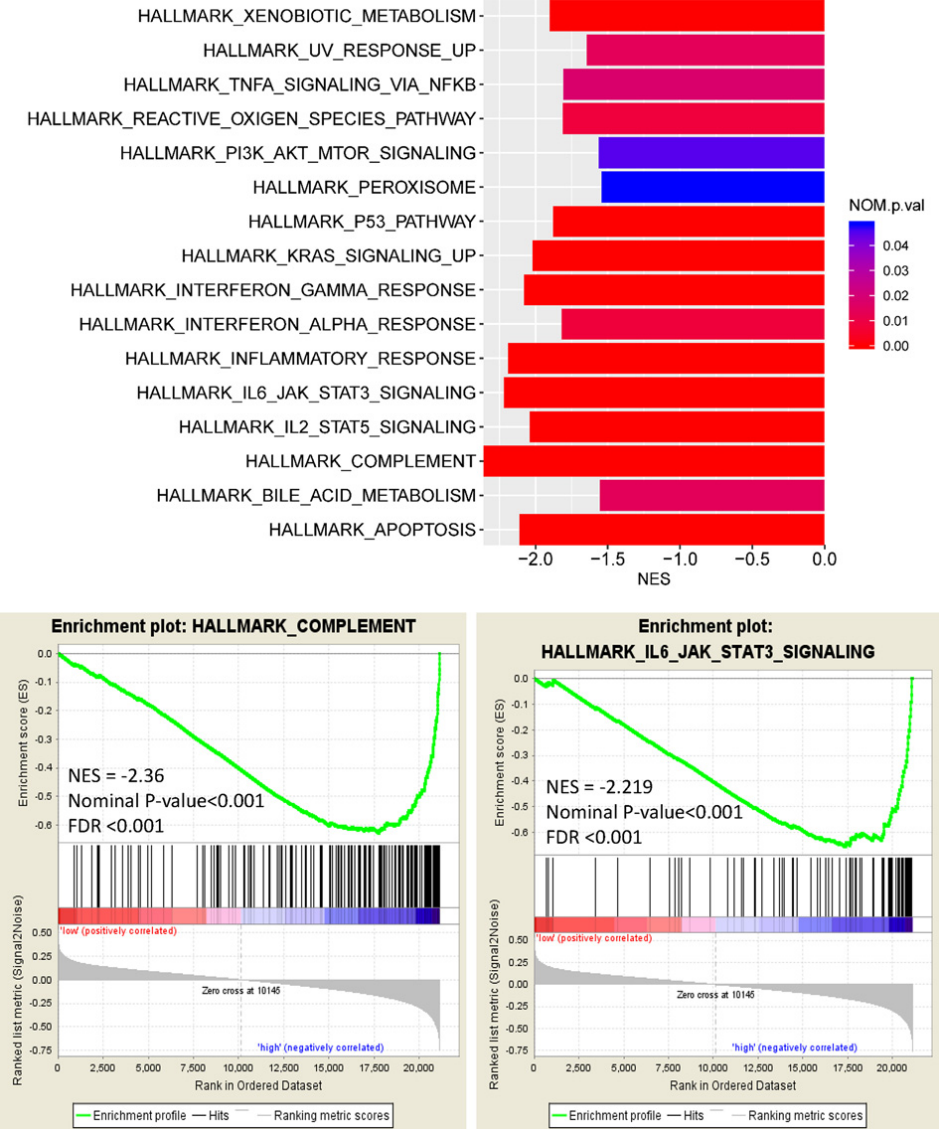
Enrichment plot from HALLMARK for CXCR3-related signaling pathways

To identify signaling pathways activated by CXCR3 in osteosarcoma prognosis, GSEA was performed. Figure 4 and Supplementary Figure S1 show significant results (FDR < 0.25, *P*-value, nominal *P*-value (NOM *P*-value) <0.05) in the enrichment of ‘h.all and c2.cp.KEGG.v6.2.symbols’. The peroxisome, apoptosis, metabolism, KRAS, P53, reactive oxygen species (ROS), PI3K/Akt/mTOR, inflammatory response, interferon-α/γ response, complement, IL-6/JAK/STAT3, IL-2/STAT5, and TNF-α via NF-κB were regulated in the high CXCR3 phenotype (Figure 4). Similarly, the gene signatures from KEGG also demonstrated that cell adhesion, apoptosis, metabolism, peroxisome, lysosome, leukocyte transendothelial migration, vascular endothelial growth factor (VEGF), calcium, MAPK, PPAR, NOTCH, natural killer cell-mediated cytotoxicity, antigen processing and presentation, B/T-cell receptor, cytokine–cytokine receptor interaction, chemokine, Fc γ receptor-mediated phagocytosis, Toll/NOD-like receptor, complement, JAK-STAT, Fc ϵ RI, and RIG-I-like receptor were also modulated in the high CXCR3 expression group (Supplementary Figure S1).

### Tumor-infiltrating immune cells

**Figure 5:**
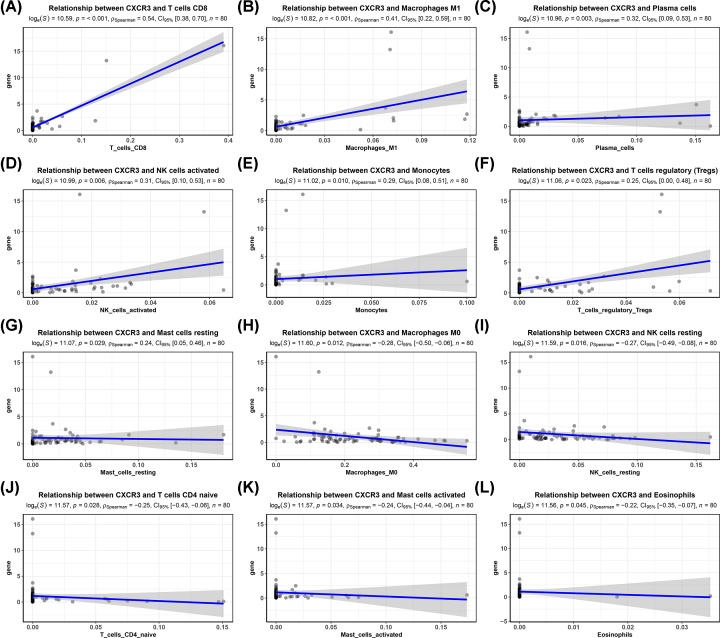
Association of CXCR3 expression with tumor-infiltrating immune cells (**A**) T cells CD8. (**B**) Macrophages M1. (**C**) Plasma cells. (**D**) NK cells activated. (**E**) Monocytes. (**F**) Tregs. (**G**) Mast cells resting. (**H**) Macrophages M0. (**I**) NK cells resting. (**J**) T cells CD4 naive. (**K**) Mast cells activated. (**L**) Eosinophils.

According to the results of GSEA, CXCR3 involved in the immune regulation such as TNF-α via NF-κB, complement, natural killer cell-mediated cytotoxicity, antigen processing and presentation, and B/T-cell receptor. Final 80 osteosarcoma samples with a CIBERSORT *P*-value of <0.05 were identified. Thus, we further analyzed whether CXCR3 expression was related to immune infiltration in 80 osteosarcomas, including 22 immune cell types. As shown in Figure 5, CXCR3 expression was positively correlated with T cells CD8 (r = 0.54, *P*<0.001), macrophages M1 (r = 0.41, *P*<0.001), plasma cells (r = 0.32, *P*=0.003), NK cells activated (r = 0.31, *P*=0.006), monocytes (r = 0.29, *P*=0.010), regulatory T cells (Tregs) (r = 0.25, *P*=0.023), and mast cells resting (r = 0.24, *P*=0.029), and was negatively correlated with macrophages M0 (r = −0.28, *P*=0.012), NK cells resting (r = −0.27, *P*=0.016), T cells CD4 naive (r = −0.25, *P*=0.028), mast cells activated (r = −0.24, *P*=0.034), and eosinophils (r = −0.22, *P*=0.045).

## Discussion

In recent years, the expression and roles of CXCRs in many cancers have been reported [7,8,14,18]. Chemokines and their receptors have crucial functions in the immune system and play essential roles in cancer development and progression [7]. CXCR3 is involved in many functions, including chemotactic migration, cell adhesion, proliferation and invasion, tumor mediating immunity, angiogenesis, and metastatic spread [11–13]. Low CXCR3 expression is associated with worse prognosis in renal cell carcinoma and gastric cancer [8,13]. To the best of our knowledge, CXCR3 expression and its potential survival impact on osteosarcoma are still not explored, the potential function of CXCR3 in regulating biological behaviors involved in osteosarcoma prognosis is further conducted.

In the present study, we observed that the expression of CXCR2, CXCR4, and CXCR5 were not correlated with the prognosis, but CXCR3 low expression was correlated with poor prognosis in osteosarcoma. Multivariate Cox analysis showed that CXCR3 remained an independent prognostic factor for predicting OS. OS was used as the most common gold standard end point in clinical studies. Because OS is easily measured, the definition is precise, and measurement is reliable and unbiased [19,20]. In this study, an event such as progression, recurrence, second malignant neoplasm, or death was defined as EFS. The definition and measurement of EFS may have a potential bias. Thus, CXCR3 did not show a statistically significant result on EFS by multivariate Cox analysis. Besides, we found that the cell adhesion, apoptosis, metabolism, KRAS, P53, NOTCH, ROS, PI3K/Akt/mTOR, VEGF, inflammation, and immune-related pathways were regulated by CXCR3. CXCR3 was associated with immune infiltration in osteosarcoma. Thus, CXCR3 may serve as a useful prognostic biomarker and could serve as a novel therapeutic target of osteosarcoma.

CXCR3 is a seven-transmembrane G-protein–coupled receptor which mediates tumor migration, invasion, angiogenesis, and immunity [21]. CXCR3 is shown to trigger several downstream pathways such as MAPK, SRC, and PI3K signaling, which may affect several cellular functions such as calcium influx, cell adhesion, proliferation, and migration [12,21]. VEGF is considered as a crucial mediator of angiogenesis and has a vital function in diverse cellular responses such as cell adhesion, survival, migration, and invasion [22]. The up-regulation of CXCR3 involves the inhibition of angiogenesis in renal cell carcinoma [23]. P53 mediates genomic stability, growth, proliferation, and immunoproperties of mesenchymal stem cells [24]. CXCR3 may induce apoptosis by the up-regulation of p53 and Bax through the p38-MAPK signaling pathway [25]. ROS have been identified as signaling molecules in a variety of pathways regulating cell survival and death, autophagy, hypoxia, genetic instability, apoptosis, angiogenesis, and T-cell immune response in the TME, which are responsible for cancer progression and resistance to therapy [26–28]. CXCR3 knockdown may prevent mitochondrial ROS accumulation in hepatocytes [29]. In this work, we observed that the peroxisome, cell adhesion, apoptosis, metabolism, KRAS, P53, ROS, PI3K/Akt/mTOR, VEGF, calcium, MAPK, PPAR, NOTCH, inflammation, and immune-related pathways such as natural killer cell-mediated cytotoxicity, antigen processing and presentation, B/T-cell receptor, cytokine–cytokine receptor interaction, chemokine, Fc γ receptor-mediated phagocytosis, Toll/NOD-like receptor, complement, JAK-STAT, Fc ϵ RI, and RIG-I-like receptor, IL-6/JAK/STAT3, IL-2/STAT5, and TNF-α via NF-κB were regulated by CXCR3 in osteosarcoma, which were the first to be reported, and the mechanisms modulated by CXCR3 need to be further elucidated in osteosarcoma.

Low CXCR3 expression was associated with unfavorable prognosis in gastric cancer [8]. CXCR3 is involved in renal cell carcinoma cell migration, invasion, and clonogenic ability [30], and decreased CXCR3 expression is correlated with worse prognosis in patients with renal cell carcinoma [13]. In this work, our study was the first to demonstrate that low CXCR3 expression was related to histologic response and worse prognosis of osteosarcoma, which is consistent with the previous studies. Although CXCR3 did not achieve a statistical significance on EFS using multivariate Cox analysis (*P*<0.05), multivariate Cox analysis showed a trend towards a correlation between low CXCR3 expression and a poor EFS (HR = 4.45, *P*=0.085). Therefore, the stratified analysis showed that low CXCR3 expression was associated with a worse EFS in female osteosarcoma patients, patients aged less than 15.1 years, and patients without metastasis. Additionally, we further explored the prognostic role of CXCR3 expression in various clinicopathological variables of osteosarcoma and found that low CXCR3 expression was still significantly associated with worse OS in females, patients aged less than 15.1 years, or patients without metastasis. The presence of high CXCR3 expression on CD8 T cells in colorectal cancer has been reported. CXCR3 is functionally expressed on Tregs in colorectal cancer [31]. A significant increase in the number of NK cells and CD8^+^ T cells expressing CXCR3 is shown in ovarian cancer [32]. Higher CXCR3 expression is correlated with increased CD8^+^ and CD4^+^ cell infiltration, and low CXCR3 expression is associated with poor prognosis in gastric cancer [33]. Our work demonstrated that CXCR3 expression was associated with tumor-infiltrating immune cells, which showed a positive association with immune infiltration of T cells CD8, macrophages M1, plasma cells, and NK cells activated. This suggested that CXCR3 played a crucial role in immune infiltration in osteosarcoma.

In conclusion, the present study demonstrated that CXCR3 in osteosarcoma was correlated with poor prognosis and immune cell infiltration. Multivariate Cox analysis further showed that CXCR3 might serve as an independent prognostic factor, especially for predicting OS. Cell adhesion, apoptosis, metabolism, KRAS, P53, NOTCH, ROS, PI3K/Akt/mTOR, VEGF, inflammation, and immune-related pathways may be the key pathways modulated by CXCR3. More clinical and experimental studies should be conducted to further validate the role of CXCR3 in osteosarcoma.

## Supplementary Material

Supplementary Figure S1 and Table S1

## Abbreviations

CIBERSORT: Cell type Identification By Estimating Relative Subsets Of known RNA Transcripts
CXCR: CXC chemokine receptor
EFS: event-free survival
ELR: glutamic acid-leucine-arginine sequence
FDR: false discovery rate
GSEA: Gene set enrichment analysis
HR: hazard ratio
KEGG: Kyoto Encyclopedia of Genes and Genomes
NOD: nucleotide-binding and oligomerization domain
OS: overall survival
PPAR: peroxisome proliferator-activated receptor
RIG-I: retinoic acid-inducible gene I
ROS: reactive oxygen species
TARGET: Therapeutically Applicable Research to Generate Effective Treatments
TME: tumor microenvironment
TPM: transcripts per kilobase million
Treg: regulatory T cell
VEGF: vascular endothelial growth factor
95% CI: 95% confidence interval
