# Supplementary material for: CXCR3 from chemokine receptor family correlates with immune infiltration and predicts poor survival in osteosarcoma
Source: Biosci Rep. 2019 Nov 12;39(11):BSR20192134. doi: 10.1042/BSR20192134 (PMC6851512; doi:10.1042/BSR20192134)
Supplement: Supplementary Figure S1 and Table S1 [file BSR-2019-2134_supp.pdf]

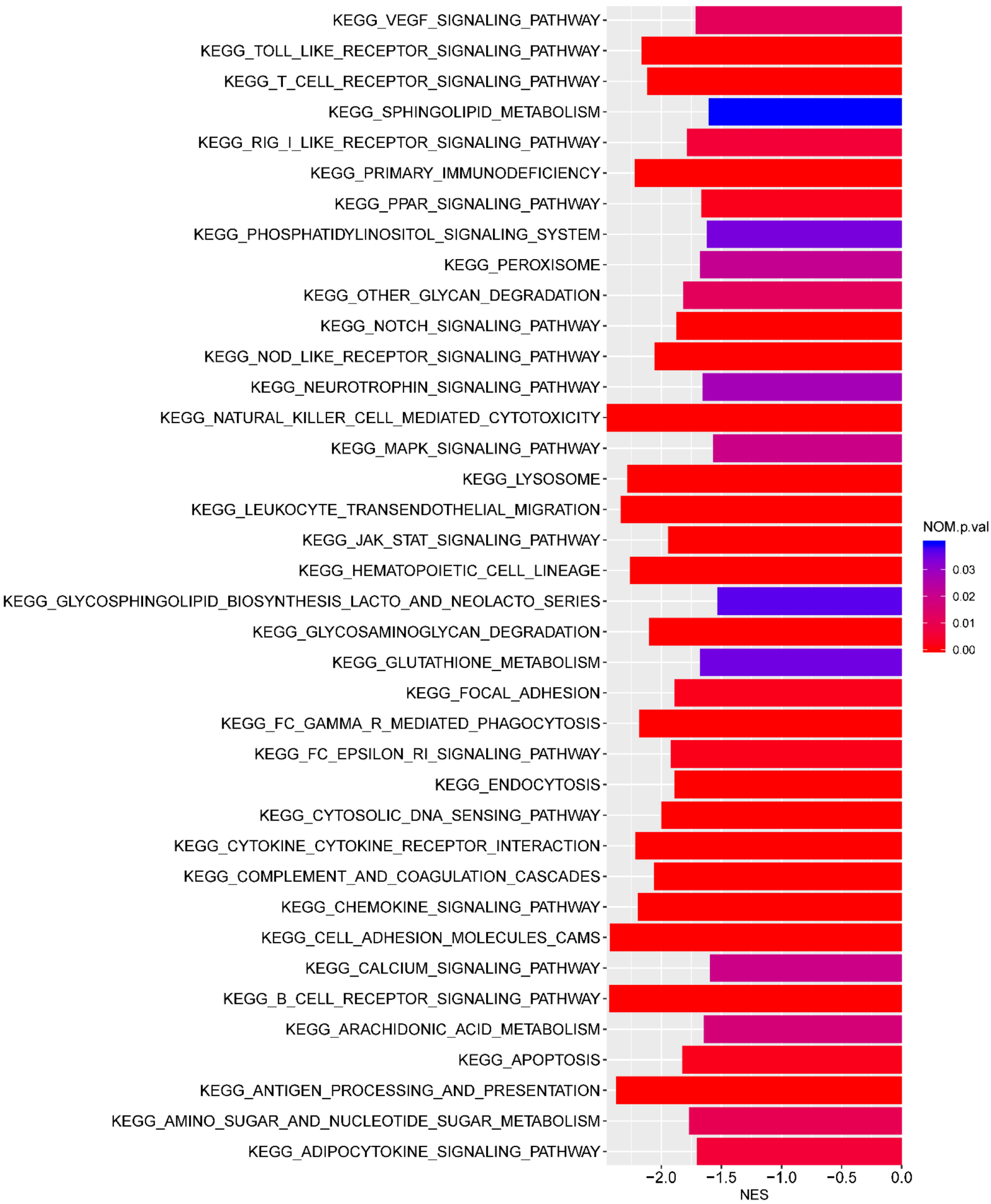

Figure S1. Enrichment plot from KEGG of GSEA for CXCR3-related signaling pathways. NOM P-value: nominal P-value; NES: normalized enrichment score.

Table S1. Basic characteristics of the study population

Characteristics		Number of cases	% (percent)
Age	Median: 15.1 years, range 3.6-39.9 years		
Gender			
	Male	58	59.2%
	Female	40	40.8%
Surgical approach			
	Limb sparing	48	87.3%
	Amputation	6	10.9%
	Limb sparing and amputation	1	1.8%
Tumor site			
	Femur	47	69.1%
	Tibia	21	30.9%
Tumor region			
	Distal	35	56.4%
	Proximal	24	38.7%
	Other, not specified/posterior	3	4.8%
Progression			
	Yes	18	37.5%
	No	30	62.5%
Histologic response			
	Poor (necrosis of ≤ 90%)	34	65.4%
	Good (necrosis of > 90%)	18	34.6%
Metastasis			
	Yes	24	24.5%
	No	74	75.5%
